# Cross-species single-cell and spatial transcriptomics map the conserved stromal remodeling landscape of inflammatory arthritis

**DOI:** 10.3389/fmmed.2026.1951540

**Published:** 2026-10-19

**Authors:** Marxa L. Figueiredo

**Affiliations:** Department of Basic Medical Sciences, College of Veterinary Medicine, Purdue University, West Lafayette, IN, United States

**Keywords:** immune cells, mouse models, rheumatoid arthritis, single-cell RNA sequencing, stromal

## Abstract

**Background:**

Rheumatoid arthritis (RA) is a condition characterized by chronic synovial inflammation and joint destruction. However, the precise networks linking cellular activation to stromal-mediated pathology remain poorly defined, hindering the development of targeted therapies.

**Methods:**

We integrated single-cell RNA sequencing (scRNA-seq) across multiple preclinical mouse models to construct a unified synovial inflammatory arthritis landscape, later validating our findings in a human RA dataset using Visium HD spatial transcriptomics.

**Results:**

Unsupervised clustering of the mouse synovium resolved distinct structural, vascular, and immune niches. Interactome mapping identified Vascular Mural cells as central hubs receiving endothelial- and mural-derived Notch signaling. Downstream of this perivascular trigger, regulatory network analysis revealed a synchronized, stress-induced Atf3/Fos (AP-1) transcription factor regulon program coordinating myofibroblast differentiation. This pathway is conserved in human RA; a homologous matrix-remodeling gene expression module (SM01, including *VIM*, *FN1*, *MMP2*) was highly active in human Activated FLS and Lining FLS. Visium HD spatial mapping physically positioned these circuitries, demonstrating that FN1 and THBS signaling networks co-localized in a highly active niche restricted to the synovial tissue’s edge.

**Conclusion:**

We define a conserved Notch/AP-1 to Fn1/THBS module regulatory axis operating within a distinct spatial niche. This model moves beyond traditional cellular profiling to identify therapeutic targets potentially useful towards simultaneously halting synovial inflammation and joint destruction.

## 1 Introduction

Rheumatoid arthritis (RA) is a chronic, systemic autoimmune disease characterized by persistent synovial inflammation, cartilage degradation, and progressive joint destruction (Isomäki and Punnonen, 1997; Zhang et al., 2023; Schett and Gravallese, 2012). Despite the successful clinical implementation of biological and small-molecule therapies targeting classical immune mediators, such as TNF-α, IL-6, and JAK/STAT pathways, a substantial cohort of patients still come to experience refractory disease or incomplete remission (Guo et al., 2018). This clinical gap thus reflects a fundamental limitation in current therapeutic paradigms. While recently approved therapeutics work at dampening systemic immune activation, they frequently fail to arrest the localized, self-amplifying stromal remodeling processes that promote joint destruction.

The synovial stroma, composed primarily of fibroblast-like synoviocytes (FLS), endothelial cells, and perivascular mural cells, has emerged as a highly active, pathological driver of RA. It used to be that this stromal compartment rather represented a passive bystander to leukocyte infiltration, but now it is recognized that synovial fibroblasts can transition into highly aggressive, myofibroblastic phenotypes. This compartment then gains the ability to abnormally express matrix metalloproteinases (MMPs), deposit aberrant extracellular matrix (ECM), and recruit immune cells through the production of sustained chemokine gradients (Turner and Filer, 2015). Gaps still remain in our understanding of what are the microenvironmental triggers associated with initiating this stromal transition, the downstream transcriptional networks that lock these cells into a pathogenic state, and the spatial architecture that might sustain these self-amplifying signaling niches.

Recent advances in single-cell RNA sequencing (scRNA-seq) have revolutionized our understanding of cellular heterogeneity within the arthritic joint, enabling the identification of various stromal and immune cell subsets (reviewed in Figueiredo (2024). Yet, single-cell datasets from individual mouse models often capture distinct immunological phases or etiology-specific pathologies (Kong et al., 2023). To overcome these limitations and construct a conserved view or landscape of the molecular programs driving synovial remodeling, we worked to integrate scRNA-seq datasets across several preclinical models of inflammatory arthritis’ synovial tissues. The convergence of these models captures diverse and complementary facets of arthritis pathogenesis. For example, Collagen-Induced Arthritis (CIA) replicates the chronic, autoimmune, and T-cell/B-cell-dependent phases of systemic joint destruction, serving as a classic model for autoimmune-driven pathology (Brand et al., 2004). In contrast, Serum Transfer-Induced Arthritis (STIA) bypasses the adaptive priming phase to mimic the acute, antibody- and innate immune-mediated effector phase of the disease. To evaluate localized, antigen-specific, T-cell-driven monoarticular joint inflammation, we integrated Antigen-Induced Arthritis (AIA) (Kehoe et al., 2014), while the Glucose-6-Phosphate Isomerase-Induced Arthritis (G6PI) model (Nandakumar et al., 2006) was incorporated to represent metabolic-immune crosstalk and rapid, systemic joint pathology. By executing a comprehensive single-cell analysis across these integrated datasets, we produced a multicellular landscape of synovial inflammation. This study identifies a highly active, conserved vascular-to-structural signaling axis relating to Notch signaling as a primary physical trigger for stromal activation. By moving past traditional cellular profiling, this work identifies relevant therapeutic targets that can potentially be utilized to halt synovial inflammation and structural joint destruction.

## 2 Materials and methods

### 2.1 Samples

We performed a comprehensive scRNA-seq analysis following integration of 12 healthy controls and 15 RA samples from publicly available datasets from various preclinical mouse models of advanced forms of arthritis (RA-focused). These datasets include GSE206651, GSE185696, and GSE224221 (collagen-induced arthritis or CIA) (Korsunsky et al., 2022; Lilja et al., 2023; Wei et al., 2020; Muench et al., 2022), GSE145286 (serum transfer-induced arthritis, STIA) (Wei et al., 2020), GSE193536 (antigen-induced arthritis, AIA), GSE184609 (glucose-6-phosphate isomerase-induced arthritis, G6PI) (Muench et al., 2022), and GSE230145 (Torres et al., 2023) and GSE129087 (CD45-negative STIA samples) (Croft et al., 2019). Our study focused on integration and characterization of the immune cell composition, as well as in identifying key differences in cellular states and dynamics across healthy and diseased state using these models.

### 2.2 Bioinformatics analyses

#### 2.2.1 Data preprocessing and quality control

Single-cell RNA-seq data were processed using two complementary platforms: Trailmaker (Parse Biosciences) (Trailmaker, 2024) for initial data handling and ICARUS v3 (Jiang et al., 2024) for downstream clustering and communication analysis. Unfiltered count matrices were uploaded, and we removed background noise using a transcripts-per-cell threshold and filtered out dead cells based on high mitochondrial content. Outliers were eliminated via linear regression, and potential doublets were filtered using scDblFinder (Germain et al., 2021). Data normalization, Principal Component Analysis (PCA), and integration with Harmony (Korsunsky et al., 2019) were performed on high-quality cells. Clusters were identified using the Leiden method, with UMAP for visualization. Cluster-specific marker genes were identified through the Wilcoxon rank-sum test via the presto package. For cell-cell communication prediction, we employed CellChat analyses.

#### 2.2.2 Differential expression and comparative analysis

To explore transcriptional differences between inflamed and healthy joints, we initially focused on genes upregulated in mouse RA_joint samples relative to healthy_joint using ICARUS to compare by sample type. These DEGs were analyzed using Metascape (Zhou et al., 2019) and STRING-DB (Szklarczyk et al., 2021; Szklarczyk et al., 2019), to uncover conserved pathways and functional modules that were potentially shared between models, with the objective of generating insights into potential mechanisms underlying the inflammatory arthritis progression among the various mouse models examined in this integration study. These DEG are presented in Supplementary Table S1. Additionally, another analysis used single cell raw counts aggregated into pseudobulk profiles by individual biological replicates (n = 15 RA vs. n = 12 healthy) for primary target clusters (clusters 1, 2, 5, 10, and 11). Differential expression analysis was performed using DESeq2. To eliminate low-count noise, counts were filtered to retain genes with ≥10 reads in at least 8 samples prior to testing, and log2 fold-changes were shrunk using the *apeglm* algorithm (Zhu et al., 2019). Statistically significant DEGs were defined using a Benjamini–Hochberg adjusted threshold of padj < 0.05. This differential expression output is provided in Supplementary Table S2.

#### 2.2.3 Clustering, intercellular signaling, and regulatory network inference

Advanced clustering and communication analyses were performed with ICARUS v3, which leverages geometric sketching to efficiently subsample and represent cellular diversity. Clustering was performed using the top 10 principal components, a k-nearest neighbor value of 10, and Louvain clustering at a resolution of 0.7, yielding 25 distinct clusters. UMAP projections were generated in raster model, ideal for visualization of many clusters. To isolate disease-specific intercellular communication, we applied CellChat (Jin et al., 2021), via a direct comparative framework between the RA disease (n = 15) and healthy control (n = 12) subsets, inferring signaling networks based on known ligand-receptor interactions. We utilized Metascape to examine the enriched pathways and processes, as well as enrichment analysis using the TRRUST database, where enriched transcription factors (-log10(P)), along with the CellChat intercellular signaling dynamics, provided a comprehensive view of the molecular interactions shaping the arthritic joint microenvironment examined. For regulatory networks, we used SCENIC (Aibar et al., 2017).

#### 2.2.4 Spatial transcriptomics processing and network analysis using VST-DAVis

Spatial transcriptomic mapping was performed on a publicly available human RA synovial tissue dataset collected prior to receiving treatment (10x Genomics Visium HD; Zenodo DOI: 10.5281/zenodo.16995699), using the Visium Spatial Transcriptomics Data Analysis and Visualization (VST-DAVis v1.0) interactive pipeline (Jagadesan and Guda, 2026). Briefly, sequencing from a single sample (400.1 million total reads) yielded 474,081 tissue-associated 8 µm bins with a mean of 197.2 UMIs per bin. Sequencing and QC metrics are provided in Supplementary Table S3. Raw Space Ranger output files (including HDF5 feature-barcode matrices and physical spatial coordinates) underwent QC filtering to remove low-quality bins with low UMI counts, low feature counts, or elevated mitochondrial gene expression. Bins passing QC were normalized and scaled using variance-stabilizing transformation. Dimensionality reduction via Principal Component Analysis (PCA) was followed by unsupervised Louvain clustering on the top principal components to define spatially resolved tissue domains. Differentially expressed spatially variable genes were identified for each domain, and spatially constrained cell-cell communication networks were inferred using CellChat to map incoming and outgoing spatial signaling pathways.

## 3 Results

### 3.1 Single-cell profiling of the inflammatory synovial microenvironment identifies distinct niches

To dissect the cellular heterogeneity and spatial compartmentalization of the joint during chronic inflammatory arthritis, we performed an integration of several single-cell RNA sequencing (scRNA-seq) datasets to compile the cellular signatures of inflammatory arthritis represented by several mouse models, as described in *Materials and Methods.* Following quality control and batch correction, unsupervised clustering and Uniform Manifold Approximation and Projection (UMAP) dimensionality reduction resolved the synovial microenvironment into three primary functional niches: a *structural/fibroproliferative* niche, a *vascular/endothelial* niche, and an *immune (myeloid/lymphoid)* niche (Figure 1). All clusters are defined in Table 1, including subclusters, with basis on analyses including the dendrogram and marker genes shown in Supplementary Figure S1 and disease vs. healthy distribution shown in Supplementary Figure S2.

**FIGURE 1:**
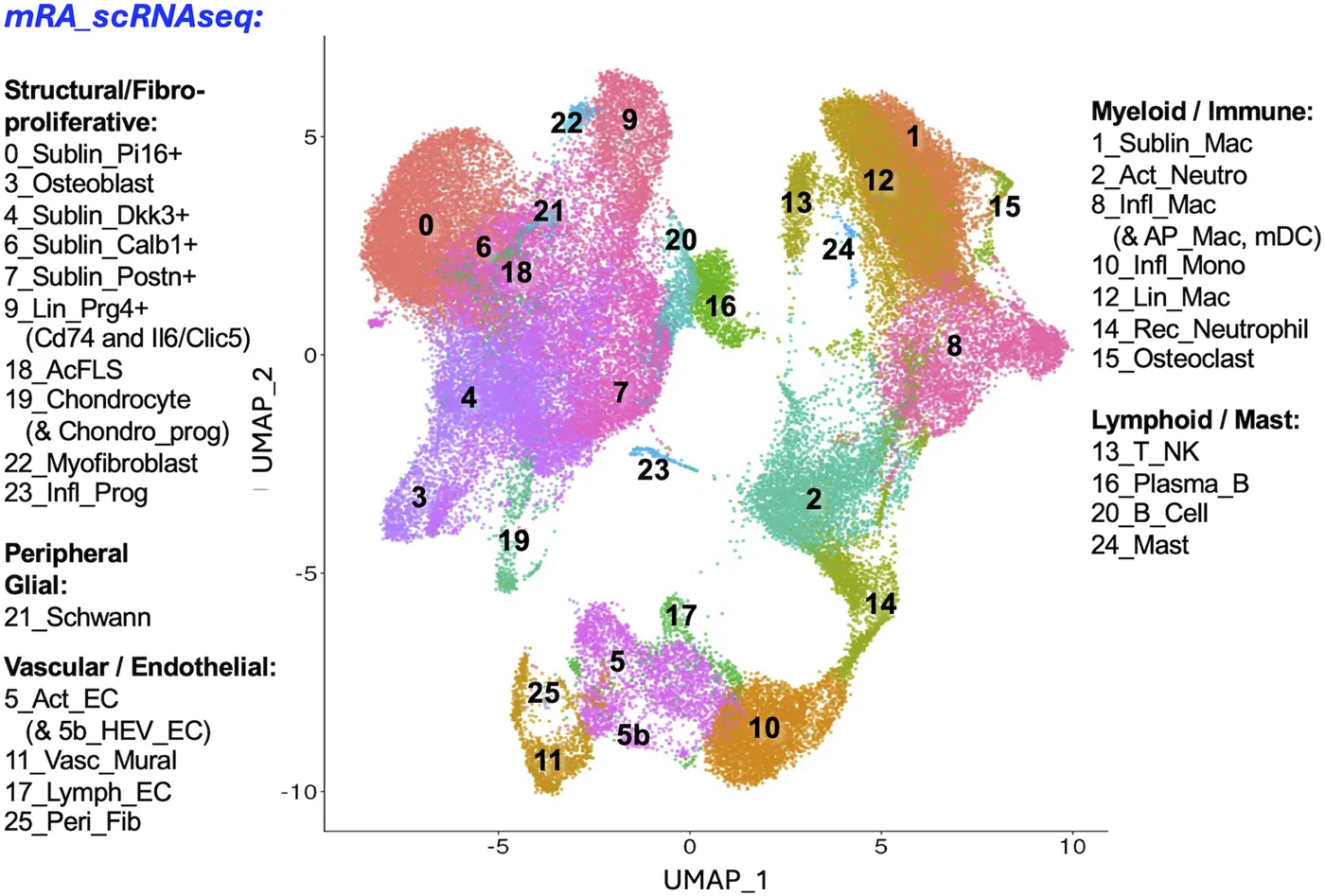
Single-cell transcriptomic profiling of the multi-model mouse synovium reveals conserved cellular taxonomy. Uniform Manifold Approximation and Projection (UMAP) of transcriptionally distinct cellular clusters integrated from a multi-model mouse rheumatoid arthritis (RA) dataset, highlighting major Structural/Fibro-proliferative, Vascular/Endothelial, Peripheral Glial, Myeloid/Immune, and Lymphoid/Mast lineages.

**TABLE 1:** Cell Clusters by Functional Compartment (mouse scRNAseq)

Compartment	Cluster	Description	Key markers
Structural/fibro-proliferative niche	9a 9b	Lining FLS Infl_lin_FLS	*Prg4, Ackr2, Sema3a, Ptger3* *Prg4, Il6, Clic5*
	0	Universal Sublining Progenitors (Sublin_Pi16+)	*Pi16, Clec3b, Gsn, C4b, Entpd2*
	6	Specialized Sublining Stromal Cells (Sublin_Calb1+)	*Calb1, Lama2, Abca8b, Adamtsl3, Npffr1*
	4	Mechanosensitive Sublining FLS (Sublin_Dkk3+)	*Dkk3, Sema3a, Snap91, Dlx3*
	7	Fibrogenic Sublining FLS (Sublin_Postn+)	*Postn, Sfrp1, Cthrc1, Mest, Ccn4*
	18	Activated FLS (AcFLS)	*Tnfsf11, Mmp3, Mmp13, Cxcl1, Cxcl2*
	22	Myofibroblasts (Myofibroblast)	*Acta2, Tagln, Pdgfrb, Ccl2, Ckm*
	23	Inflammatory Progenitors (Infl_Prog)	*Mki67, Top2a, Pcna, Stmn1, Krt14*
	19	Chondrocytes (& Chondro progenitors)	*Col2a1, Col9a1, Col9a2, Acan, Sox9, Mia*
	3	Osteoblasts (Osteoblast)	*Gphbp1, Cilp, Aqp7, Fmod, Spp1, Bglap*
Vascular/endothelial niche	11	Vascular Mural Cells (Vasc_Mural)	*Tagln, Lmod1, Acta2, Myh11, Rgs4, Notch3*
	25	Perivascular Fibroblasts (Peri_Fib)	*Plau, Ccl19, Colec11, Vcan, Plp1*
	17	Lymphatic Endothelial (Lymph_EC)	*Prox1, Ccl21a, Flt4, Mmrn1, Egfl7*
	5	Activated Endothelial (Act_EC)	*Pecam1, Cdh5, Kdr, Sele, Vcam1, Ccl2*
	5b	Venular Endothelial (HEV-like)	*Lifr, Icam1, Madcam1, Glycam1*
Myeloid/immune niche	12	Resident Lining Macrophages (Res_Mac)	*Mrc1, Msr1, Cx3cr1, Ccr5, MerTK*
	1	Sublining Synovial Macrophages (Sublin_Mac)	*MerTK, Folr2, C1qa, C1qb, Pf4*
	8 8b	Inflammatory Macrophages (Infl_Mac) Antigen presenting Mac (AP_Mac) & mDC	*Cd74, H2-Ab1, H2-Aa, Fcgr1, Il1b*
	10	Inflammatory Monocytes (Infl_Mono)	*Ccr2, Il1b, Csf3r, Ly6c1, S100a8*
	15	Osteoclasts (Osteoclast)	*Acp5, Ctsk, Calcr, Oscar, Tnfrsf11a*
	2	Activated Neutrophils (Act_Neutro)	*S100a8, S100a9, Mpo, Lcn2, Hba-a1*
	14	Resting/Recruited Neutrophils (Rec_Neutro)	*Itgb2l, S100a8, Ltf, Mmp8, Ly6g, Cxcr2*
Lymphoid/mast niche	13	T / NK Cells (T_NK)	*Cd3e, Cd3g, Trac, Trbc1, Icos, Nkg7*
	16	B Cells (B_Cell)	*Ms4a1, Cd19, Cd79a, Cd79b, Pax5*
	20	Plasma Cells / Proliferative B (Plasma_B)	*Cdca3, Mki67, Pbk, Pimreg, Igkc, Sdc1*
	24	Mast Cells (Mast_Cell)	*Tpsab1, Mcpt4, Cpa3, Mrgprb2, Ckit*
Peripheral glial niche	21	Schwann Cells (Schwann)	*Sox10, Plp1, Mpz, Drp2, Cdh19*

Within the *structural and fibro-proliferative* niche, single-cell profiling resolved a broad range of specialized stromal states, including universal progenitor-like Pi16+ sublining fibroblasts, specialized Calb1+ sublining stromal cells, mechanosensitive Dkk3+ sublining FLS, fibrogenic Postn+ sublining FLS, activated FLS, myofibroblasts, inflammatory progenitors, and activated, joint-destructive Prg4+/Il6+ lining FLS, in addition to specialized osteoblast and articular chondrocyte lineages (Figure 1A). *Vascular and endothelial* compartments included activated endothelial cells, Lifr+/Icam1+ venular endothelial cells, perivascular fibroblasts, vascular mural cells, and lymphatic endothelial cells. The *myeloid immune compartment* included protective resident lining macrophages and sublining synovial macrophages (characterized by MerTK, Selenop, Lyve1, Cx3cr1, and C1qa), and also inflammatory macrophages, recruited inflammatory monocytes, bone-resorbing osteoclasts, as well as both recruited and activated neutrophil populations. Finally, adaptive lymphoid, mast, and neural populations were represented by some populations of T/NK cells, B cells, plasma cells, mast cells, and peripheral Schwann cells.

### 3.2 Transcriptional dynamics and functional enrichment in inflammatory arthritis

To define the pathological molecular programs present in this integrated mouse model dataset of chronic joint inflammation, we performed differential expression analysis comparing inflamed joints to healthy control joints (Figure 2; Supplementary Table S1). Using a significance threshold of |Log2 FC| > 0.5 and an adjusted p-value threshold of padj < 0.05 (-log10 (p-value) > 1.3), volcano plot analysis revealed a pronounced upregulation of transcriptional programs in RA joints relative to downregulated genes (Figure 2A). Pathway enrichment analysis of these upregulated differentially expressed genes (DEGs) highlighted strong activation of innate immune cascades (*neutrophil degranulation, platelet activation and aggregation*), and extracellular matrix (ECM) signaling (*ECM organizatio*n, *supramolecular fiber organization*) (Figure 2B). Additional prominent disease pathways included *focal adhesion, regulation of proteolysis*, *IL-17A signaling*, and *positive regulation of tumor necrosis factor (TNF) production*. Protein-protein interaction and MCODE network analysis further identified interconnected functional modules focused on *collagen formation, ECM organization, response to external stimuli, regulation of actin filament organization*, and *rRNA metabolic processes* (Figure 2C). These findings demonstrate a coordinated transcriptional shift toward immune cell activation, persistent inflammatory cytokine signaling, and extensive ECM remodeling within the arthritic joint.

**FIGURE 2:**
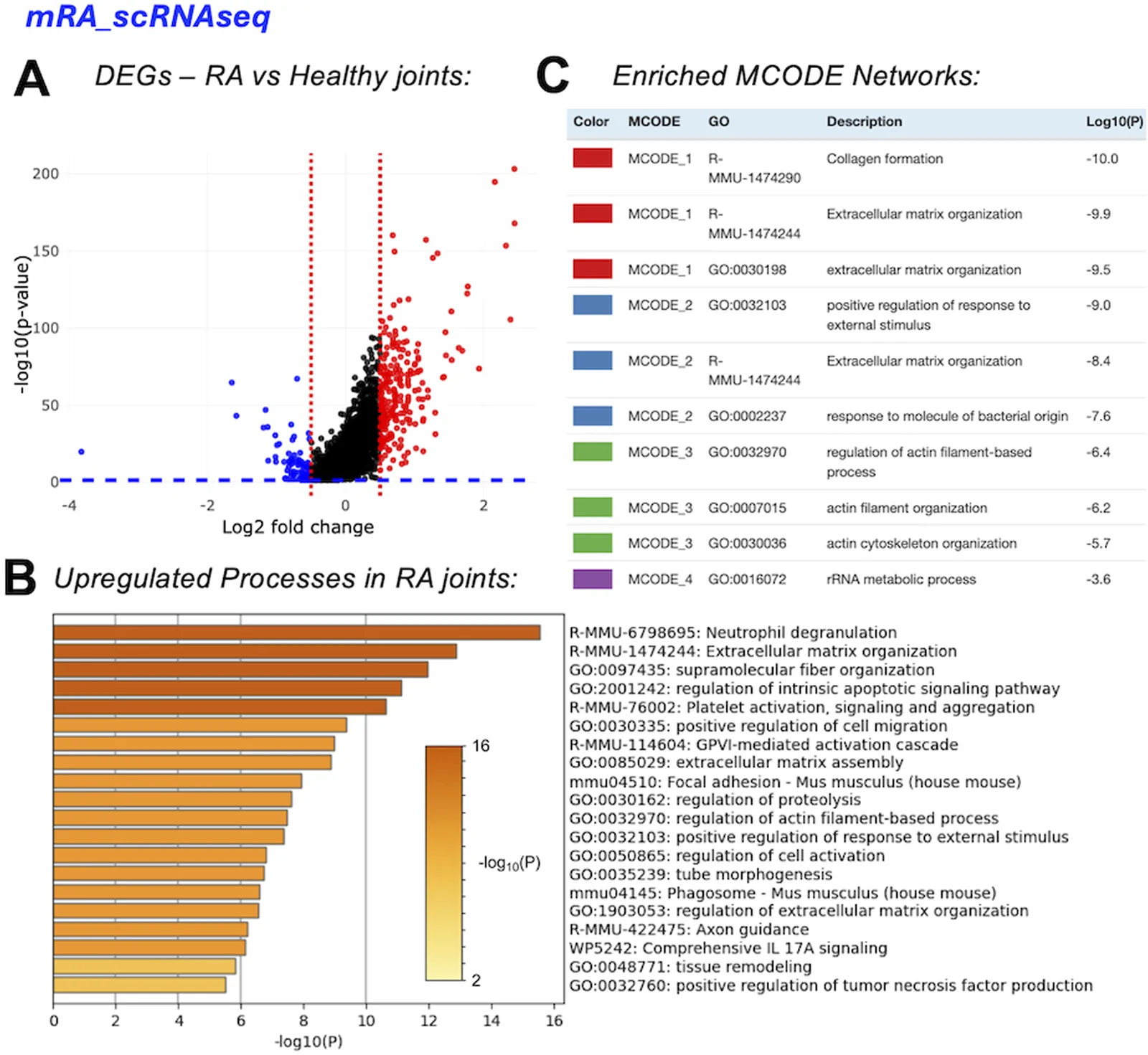
The disease-state synovial transcriptome is dominated by structural extracellular matrix remodeling. **(A)** Volcano plot depicting differentially expressed genes (DEGs) in RA versus healthy synovial tissue, highlighting differential transcriptomic upregulation in the diseased state. **(B)** Gene Ontology (GO) and pathway enrichment analysis of upregulated genes, demonstrating top functional enrichments color bar, -log10(p) values. **(C)** Molecular Complex Detection (MCODE) network analysis lists indicate the top functional interactome clusters driving pathology-upregulated matrisome and structural proteins.

Conversely, pathway enrichment analysis of downregulated DEGs revealed a loss of homeostatic tissue development and structural maintenance programs, enriched in response to wounding, developmental growth, blood vessel development, glial cell fate specification, biomineral tissue development, and fatty acid metabolic processes (Supplementary Figure S3A). MCODE network analysis confirmed suppression of functional clusters tied to baseline *blood vessel development, leukocyte migration regulation, post-translational phosphorylation, regulation of insulin-like growth factor (IGF) transport by binding proteins*, and *biological oxidations* (Supplementary Figure S3B). Our findings suggest a specific and coordinated transcriptional shift in inflammed joints away from baseline metabolic and structural tissue homeostasis toward aggressive immune cell activation, persistent inflammatory cytokine signaling, and pathological matrix remodeling.

### 3.3 CellChat interactome mapping identifies vascular mural cells and sublining fibroblasts as synovial network hubs

To identify the ligand-receptor candidates contributing to joint remodeling in inflammatory arthritis, we performed cell-cell communication analysis using CellChat on the mouse scRNA-seq dataset (Figure 3). Outgoing and incoming interaction strength and differential interaction analysis revealed a selective surge in communication within the structural and mural compartments in RA joints, with some macrophage and monocytic populations serving as prominent targets of incoming communication from these activated subsets (Figure 3A). Sublining Progenitors (Sublin_Pi16+ Cd34+), Fibrogenic Sublining FLS (Sublin_Postn+), Vascular Mural Cells, and Lining FLS (Lin_Prg4 Cd74+ and Prg4 Il6+) emerged as strongly upregulated signaling hubs in the arthritic joint, displaying the highest differential number of interactions and overall incoming/outgoing signaling strength (represented by positive red enrichment scores, Figure 3A).

**FIGURE 3:**
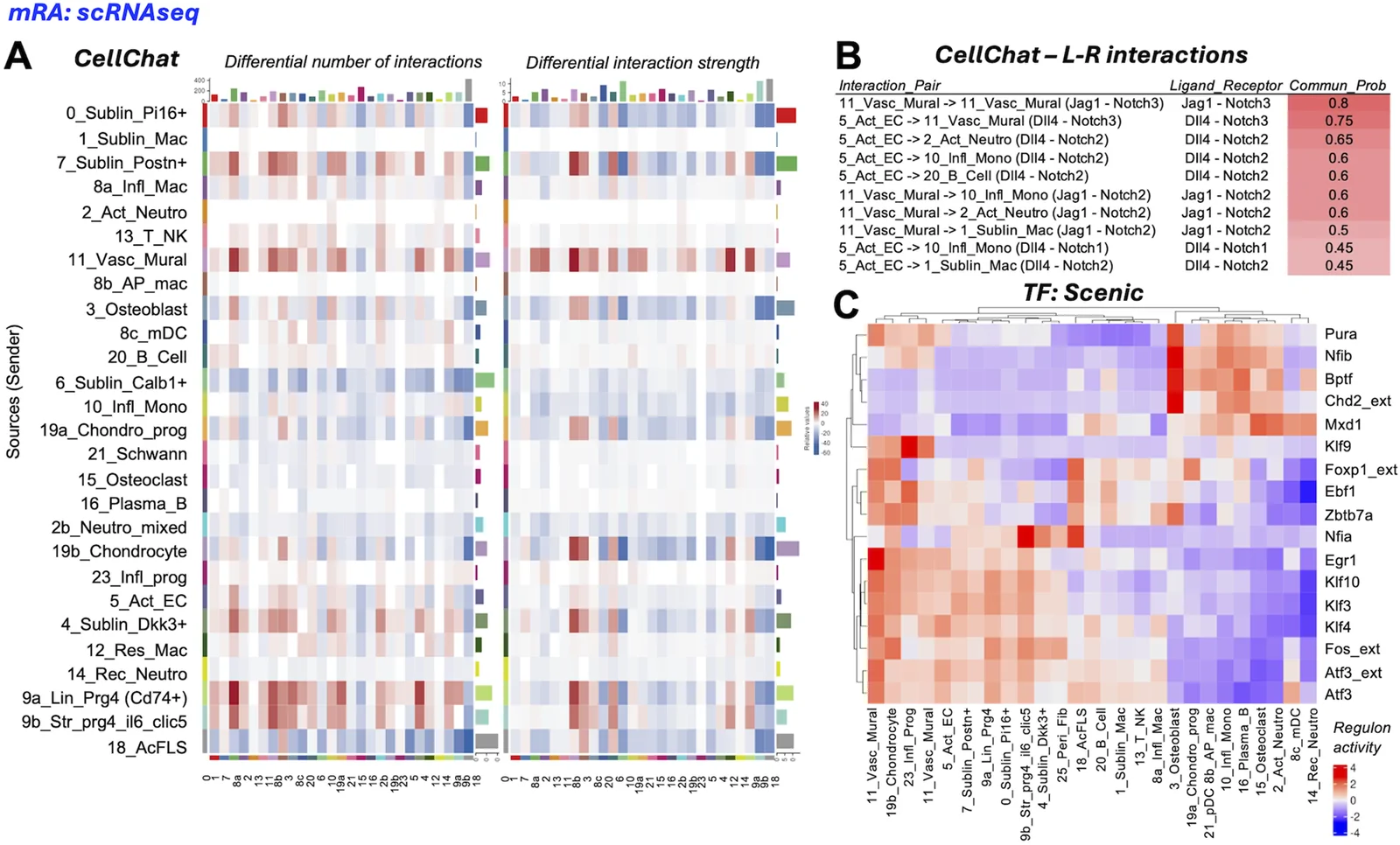
Endothelial-to-mural Notch signaling drives an Atf3-regulated transcription factor program in pathogenic stroma. **(A)** CellChat differential interaction analysis showing global incoming and outgoing cell-cell communication strength across synovial subsets in health versus disease. **(B)** CellChat ligand-receptor interaction table highlighting specific upregulated Notch pathway axes. **(C)** SCENIC (Single-Cell Regulatory Network Inference and Clustering) heatmap displaying regulon activity, demonstrating specific activation of Atf3 and Fos transcriptional networks within Vascular Mural Cells, Inflammatory progenitors, and sublining stromal populations.

Given the marked upregulation of vascular compartments as primary communication nodes, we examined specific Notch family ligand-receptor interactions across structural, vascular, and infiltrating immune niches in the arthritic joint (Figure 3B). CellChat probability modeling identified active signaling axes dominated by Jag1 and Dll4 ligands. Structurally, autocrine Jag1-Notch3 signaling within Vascular Mural Cells and endothelial-to-mural Dll4-Notch3 communication (Act_EC → Vasc_Mural) represented the highest-strength signaling links in the disease state. Beyond structural feedback, Activated Endothelial cells and Vascular Mural cells operated as key upstream senders of inflammatory cues via Notch receptors. Specifically, mural Jag1-Notch2 signaling (Vasc_Mural) maintained strong communication with infiltrating inflammatory monocytes (Infl_Mono) and sublining macrophages (Sublin_Mac). Similarly, endothelial cells directed inflammatory signals via Dll4-Notch2 to Infl_Mono, Sublin_Mac, and neutrophils, together with a Dll4-Notch1 interaction targeting Infl_Mono. In combination, these interactome dynamics suggest that while Notch3 signaling serves as a high-probability hub maintaining the vascular/mural niche, active joint inflammation utilizes a Notch1/2-dependent signaling axis to bridge activated endothelial and mural cells directly to resident and recruited synovial macrophage/monocyte populations. This positions the vascular/mural unit as an actionable upstream trigger driving both structural remodeling and macrophage activation in the inflamed synovium.

To investigate the downstream regulatory programs operating within these activated cell populations, we reconstructed single-cell gene regulatory networks using SCENIC (Figure 3C), and also verified cluster-specific consistency via replicate-aware pseudobulk DESeq2 modeling (Supplementary Table S2). Hierarchical clustering of transcription factor regulon activity revealed synchronized regulatory modules across both structural and myeloid lineages. Within Vascular Mural cells, Chondrocytes, Inflammatory Progenitors, specialized sublining subsets, and some macrophage subsets, displayed activity for Atf3 and Fos, along with Egr1 and Klf regulons. Because these TFs regulate inflammatory stress responses, this shared transcriptional module highlights a synchronized regulatory state coordinating both stromal remodeling and macrophage activation within the arthritic joint.

### 3.4 Translation to human RA identifies high activity of conserved matrix remodeling and notch programs in inflammatory stroma

To determine if the stress-induced, Notch-responsive pathway or similar regulons for ECM signature gene activation are conserved in human pathology, we analyzed a human RA spatial single-cell dataset (Visium HD). We mapped major cell lineages across the human synovium (Figure 4A) and evaluated their transcriptional profiles (Figure 4B). Heatmap analysis confirmed conserved cell-type-specific marker signatures across the human synovial niches (Figure 4B). Specifically, vascular mural lineages separated into two sub-clusters: vascular mural cells were enriched for NOTCH3, ACTA2, TAGLN, and basement membrane/collagen genes like COL4A1, whereas perivascular/adventitial stromal cells uniquely expressed high baseline levels of smooth muscle contraction and perivascular structural markers, including MYH11, MCAM, PLN, TBX2, and IGFBP3. In contrast, the FLS lineages showed distinct functional compartmentalization: Activated FLS were enriched for joint-destructive proteases, inflammatory mediators, and tissue-remodeling drivers including MMP1, MMP3, S100A8, TWISTNB, and CEMIP. This population was distinct from the classic lining FLS and inflammatory lining FLS, which were marked by strong co-expression of PRG4, CRTAC1, PCSK6, and THBS4, as well as pathogenic sublining stromal populations characterized by heavy ECM deposition markers including COL1A1, ASPN, COMP, THBS2, and FNDC1 (Figure 4B).

**FIGURE 4:**
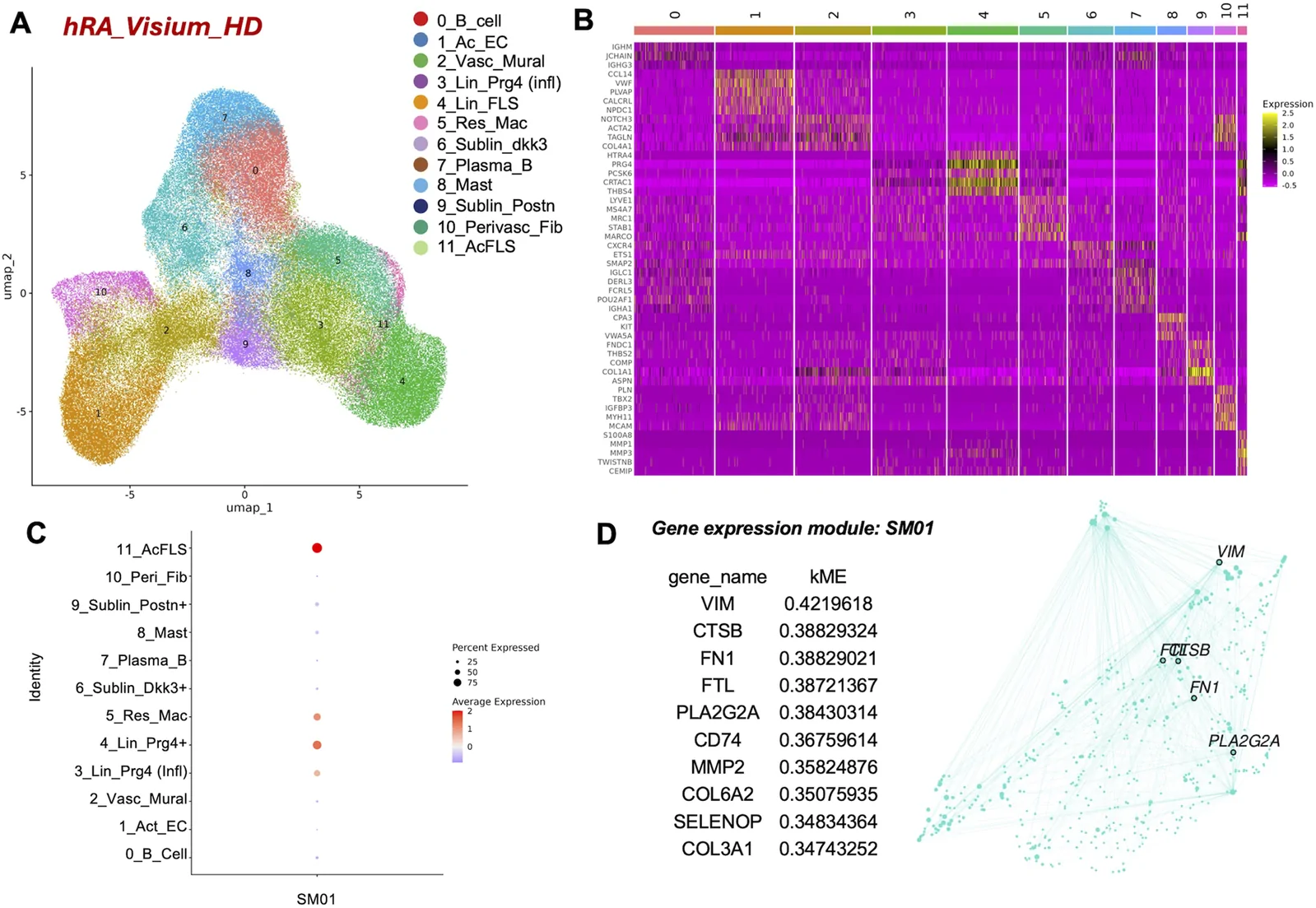
Spatial mapping of human RA synovium resolves distinct cellular lineages and matrix-remodeling modules. **(A)** Spatial UMAP mapping of transcriptomic cell lineages in human rheumatoid arthritis (hRA Visium HD). **(B)** Heatmap detailing lineage-defining gene marker expression across human synovial clusters, including Vasc_Mural, Lin_Prg4 (infl), Lin_FLS, Perivasc_Fib, and AcFLS. **(C)** Dot plot projection of the human homologous SM01 WGCNA gene co-expression module score, demonstrating selective enrichment in AcFLS (Cluster 11), Lin_FLS Prg4+ (Cluster 4), and Lin_Prg4+ Inflammatory (Cluster 3). **(D)** Module membership (kME) table and co-expression network detailing core matrix-remodeling hub genes driving a SM01 module program.

To evaluate gene regulation, WGCNA coexpression returned 1 module (SM01) (Figure 4C). Genes within this module were related to stress, structural, and matrix-remodeling hub, with high membership scores, including VIM, CTSB, FN1, FTL, PLA2G2A, CD74, MMP2, COL6A2, SELENOP, and COL3A1 (Figure 4D). Analysis of this SM01 module activity further revealed a selective enrichment in human Activated FLS, accompanied by notable expression in Lining FLS (Lin_Prg4 and inflammatory Lin_Prg4), representing both the highest average expression and highest proportion of expressing cells. These human populations represent functional and transcriptional homologs of the mouse sublining fibroblast, vascular mural, and lining FLS hubs, suggesting that the Atf3/Fos stress-response pathway and its downstream matrix remodeling program may be conserved drivers of the inflammatory stromal phenotype in human RA.

### 3.5 High-resolution spatial transcriptomics maps the structural-vascular inflammatory niche in human RA

We then leveraged the high-resolution hRA spatial transcriptomics dataset (Visium HD) to map the physical architecture of these communication networks directly within human RA synovial tissue (Figure 5). Spatial incoming and outgoing signaling pattern analysis identified Activated FLS as a central spatial coordinator in the tissue, acting as the primary source of outgoing Collagen, FN1 (Fibronectin), Tenascin, and THBS (Thrombospondin) ligands, while simultaneously remaining highly receptive to incoming NOTCH, THBS, and FN1 signaling pathways (Figure 5A). The spatial chord diagrams resolved the cell-type-specific directional communication circuits within the tissue. We confirmed Perivascular Fibroblasts presenting JAG1 signal to NOTCH3 on Vascular Mural cells (Figure 5B). Concurrently, a multi-cellular structural network mediated by THBS2, THBS4, and COMP linked Lining FLS and Activated FLS to receptor partners SDC1 and CD47 on Plasma Cells and Activated FLS. Spatial overlay maps revealed that the FN1 and THBS signaling networks co-localize at high density within the tissue architecture (Figure 5C). The highest intensity of these active signaling pathways concentrated along an active inflammatory niche at the tissue’s edge, heavily enriched for Activated FLS, Lining FLS, and Perivascular Mural cells. These spatial transcriptomic findings suggest that stress-induced, Notch-driven signaling and matrix-remodeling networks organize into a localized structural-vascular niche that coordinates chronic synovial inflammation in human RA.

**FIGURE 5:**
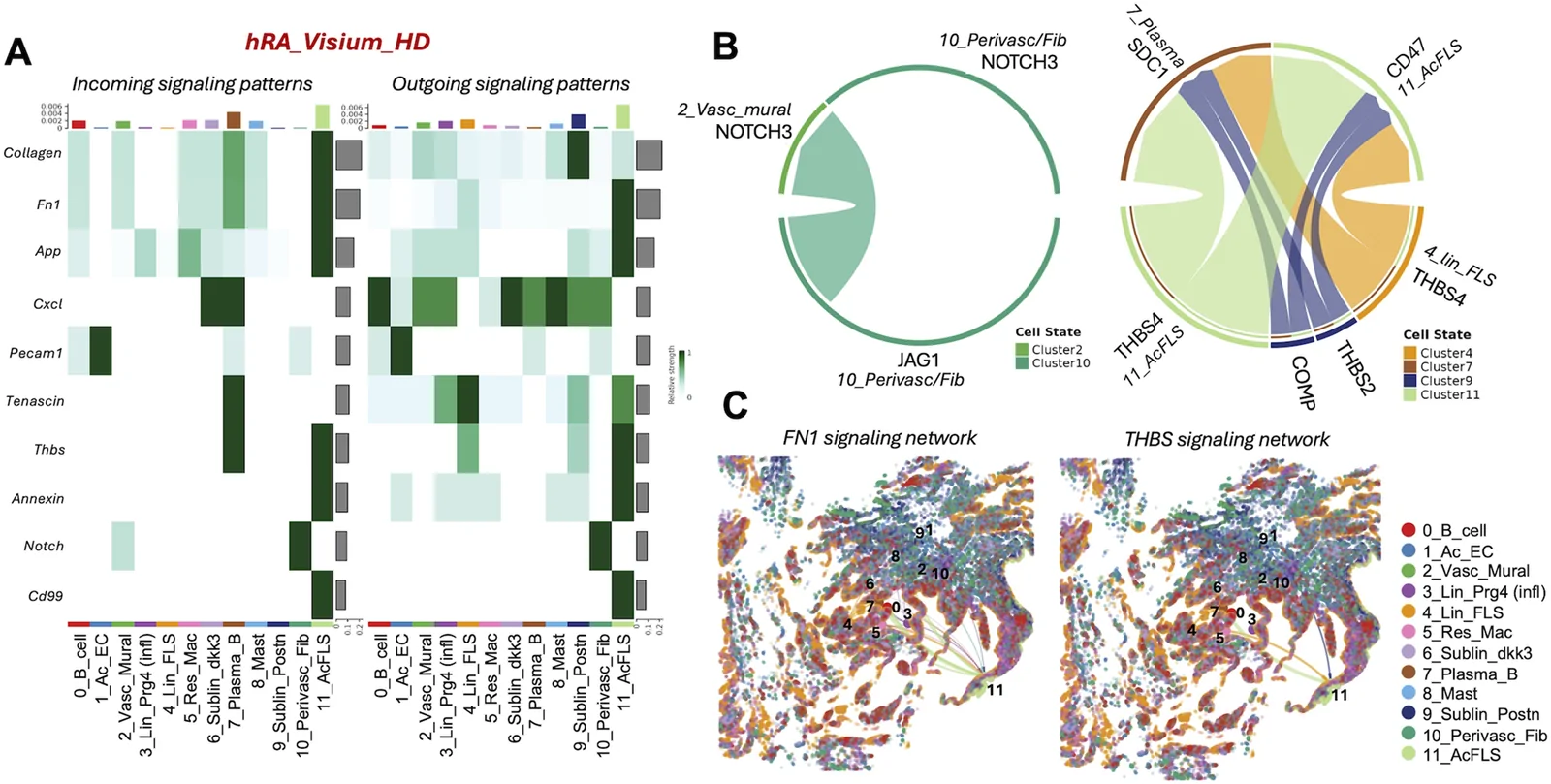
High-resolution spatial transcriptomics maps the structural-vascular inflammatory niche in human RA. **(A)** Spatial CellChat incoming and outgoing communication pattern heatmaps across human spatial transcriptomic clusters (hRA_Visium_HD), implicating AcFLS (Cluster 11) as a major hub for incoming and outgoing Collagen, Fn1, Tenascin, and Thbs signaling. **(B)** Directional spatial chord diagrams illustrating active perivascular JAG1-NOTCH3 signaling axes and also a multi-cellular THBS2/THBS4/COMP signaling network targeting SDC1 and CD47 on Plasma cells and Inflammatory stromal/FLS populations. **(C)** High-resolution Visium HD spatial overlay maps of FN1 and THBS signaling networks, suggesting dense accumulation and co-localization along the inflammatory tissue niche.

## 4 Discussion

This study presents a cross-species, spatially resolved single-cell transcriptomic analysis aiming to decode the cellular landscape and signaling networks of inflammatory arthritis, with a focus on RA. By integrating mouse models of inflammatory arthritis with human single-cell and high-resolution spatial transcriptomics, we aimed to bridge the precision gap across technologies and models to better inform therapy strategies for the complex synovial microenvironment (Mashkoor et al., 2026).

We identified specialized stromal subsets (including Sublining Pi16+, Dkk3+, and Postn+, Inflammatory Progenitors, and Lining FLS) and also specialized osteoblasts, chondrocytes, and endothelial cells. Bridging these structural and vascular niches were prominent Vascular Mural and Perivascular Fibroblast populations. This spatial compartmentalization revealed a model whereby functional cooperation drives synovial pathology in RA, consistent with recent literature (Bhamidipati et al., 2026). Beyond mapping these cellular niches, our interactome analysis revealed that vascular/perivascular and stromal subsets serve as key upregulated communication hubs. Although our findings centered on stromal networks, these populations operate in continuous crosstalk with resident and recruited synovial macrophages, providing critical survival factors, chemokines, and spatial cues that sustain macrophage-mediated joint inflammation. Crucially, we identified a high-strength perivascular Notch signaling axis, anchored by autocrine Jag1-Notch3 in Vascular Mural Cells and endothelial-to-mural Dll4-Notch3 signaling. In parallel, Activated Endothelial and Vascular Mural Cells direct inflammatory signals via Dll4-Notch2 and Jag1-Notch2 to infiltrating myeloid populations. This pathway thereby may act to instruct synovial macrophage activation and polarization within the perivascular niche. Our findings suggest that perivascular Notch signaling functions as both an upstream trigger for structural cell activation and a vascular gateway likely coordinating pathogenic macrophage recruitment into the synovial niche, offering promising therapeutic targets in human and murine RA.

Downstream of this perivascular Notch trigger, single-cell regulatory network identified a coordinated, stress-induced transcriptional program. We observed a cell-type-specific regulon cluster defined by the synchronized activity of Atf3 and Fos regulons, shared across Vascular Mural Cells, Inflammatory Progenitors, Articular Chondrocytes, specialized sublining several stromal subsets and macrophage populations. This AP-1 activation program clustered with the immediate-early gene Egr1 and Krüppel-like factors Klf4 and Klf10. These transcription factors usually can cooperate (Tallack et al., 2010; Rohini et al., 2018), suggesting that they may function as a unified regulatory hub that drives myo/fibroblast differentiation and may coordinate the aggressive, fibrotic response characteristic of chronic joint disease. The broader transcriptional profile (DEGs up and down) in the arthritis joint revealed a distinct dual imbalance. Further, the sample-level pseudobulk DESeq2 analysis across our key target clusters confirmed that these cluster-specific differential patterns mirror the global data trends from ICARUS, supporting the findings also under a replicate-aware testing framework. The upregulated transcriptional program is strongly enriched for processes driving joint destruction, including neutrophil degranulation, ECM organization, supramolecular fiber organization, platelet activation, focal adhesion networks, and IL-17A and TNF signaling. Conversely, downregulated programs were enriched in essential homeostatic pathways, including response to wounding, developmental growth, blood vessel development, glial cell fate specification, biomineral tissue development, and fatty acid metabolic processes, in agreement with the literature (Isomäki and Punnonen, 1997; Zhang et al., 2023; Turner and Filer, 2015; Nandakumar et al., 2006; Wei et al., 2020). This indicates RA pathogenesis is driven not only by the hyperactivation of destructive inflammatory pathways but also by the failure to sustain protective and regenerative programs.

To establish the translational relevance of these findings, we compared our murine observations with a human visium RA dataset. The human dataset was regulated by one key gene co-expression module (SM01), containing several core matrix-remodeling genes. Heatmap analysis confirmed conserved cell-type-specific signatures, where human mural lineages separated into Perivascular Mural cells (marked by NOTCH3, ACTA2, TAGLN) and Perivascular Fibroblasts. In parallel, human AcFLS and Lining FLS exhibited high enrichment for the SM01 module and joint-destructive proteases, suggesting the mouse model represents in many aspects the human stroma-mediated joint pathology. High-resolution spatial transcriptomics validated some of the cellular networks. In these networks, incoming and outgoing signaling patterns positioned human AcFLS as central spatial senders of COLLAGEN, FN1, TENASCIN, and THBS pathways, while remaining highly receptive to incoming NOTCH, THBS, and FN1 signals. Spatial chord diagrams confirmed active perivascular JAG1-NOTCH3 signaling between stromal cells expressing NOTCH3 and Perivascular Mural cells, alongside a multicellular matrix network (THBS/COMP) targeting SDC1 and CD47 on Plasma cells and AcFLS. High-resolution spatial overlay maps confirmed that FN1 and THBS networks co-localize within an active inflammatory niche restricted to the tissue’s edge, populated by AcFLS, Lining FLS, and Perivascular Mural cells. Despite these interesting insights, the study has notable limitations. First, while the spatial transcriptomics dataset provides high-resolution mapping from an untreated RA joint tissue, it is derived from a single human donor, serving as a proof-of-concept bridge that in future studies will benefit from broader validation. Second, CellChat analysis infers potential microenvironment signaling rather than confirming direct physical cell-cell contact *in vivo*. Consequently, future functional assays are necessary to validate these predicted pathways.

In combination, these findings provided integrative insights into the cellular and molecular alterations driving pathology in the arthritic joint. A combined mouse/human model emerges where vascular-derived Jag1 and Dll4 activate Notch3/2 on adjacent stromal and immune cells, initiating an Atf3/Fos transcriptional cascade that drives both myeloid activation and a matrix-remodeling program within a localized, self-amplifying spatial niche at the tissue’s edge. This model points toward a promising, stroma- and vascular-directed therapeutic paradigm in RA. Unlike conventional immunomodulatory biologics that broadly suppress systemic immunity, selective blockade of Notch3/2 activation or endothelial ligand presentation could specifically uncouple vascular-to-stromal and vascular-to-immune crosstalk, preventing both pathogenic FLS activation and immune infiltrate recruitment. Furthermore, disrupting this structural trigger could destabilize the self-amplifying spatial niche at the tissue’s edge. Combining perivascular Notch pathway inhibition with downstream targeted therapies against matrix-remodeling networks (THBS or FN1) or AP-1 transcription factors (Atf3/Fos) may represent a promising synergistic strategy to simultaneously halt chronic inflammation and structural joint destruction.

## 5 Conclusion

This study worked to develop a cross-species, spatially resolved framework to decode the cellular dynamics driving RA. By integrating mouse single-cell with human spatial transcriptomics, we resolved the joint microenvironment into functional niches. Our model suggests a conserved vascular signaling axis where perivascular-derived Jag1 and Dll4 trigger Notch3/2 on adjacent mural, sublining fibroblast, and synovial macrophage subsets, and we could detect also a synchronized Atf3/Fos transcriptional program. This regulatory cascade directly coordinates the expression of the human-homologous matrix-remodeling module within a very active spatial niche at the synovial tissue’s edge, heavily enriched for AcFLS, Lining FLS, and Perivascular Mural cells. Targeting these upstream perivascular mechanisms and their connections to strong downstream matrix-remodeling modules may offer a multi-tier therapeutic strategy to prevent both chronic inflammation and structural tissue damage in RA.

## Data Availability

The original contributions presented in the study are included in the article/Supplementary Material, further inquiries can be directed to the corresponding author.

## Supplementary material

SUPPLEMENTARY FIGURE S1 Comprehensive integration and interactome mapping across the multi-model mouse synovial database. Network interactome map detailing key lineage-defining marker gene networks across integrated mouse synovial cell clusters, distinguishing structural, vascular, myeloid, lymphoid, mast, and peripheral glial lineages. Color bar, log2 Fold Change.

SUPPLEMENTARY FIGURE S2 Uniform Manifold Approximation and Projection (UMAP) of healthy and RA-affected synovia. Integrated single-cell transcriptomic profiles comparing healthy control joints (n = 12, orange) versus RA-affected disease joints (n = 15, cyan), displaying relative cellular representation and structural overlap across integrated preclinical models).

SUPPLEMENTARY FIGURE S3 Differential transcriptomic profiling and pathway inactivation in the diseased synovium. **(A)** Gene Ontology (GO) enrichment analysis of downregulated genes in RA versus healthy synovial tissue, showing a significant loss of homeostatic programs including response to wounding, blood vessel development, extracellular matrix organization, biomineral tissue development, and fatty acid metabolic processes. color bar, -log10(p) values. **(B)** Molecular Complex Detection (MCODE) network analysis table showing interactome clusters (including regulation of epithelial cell proliferation, response to wounding, blood vessel development, leukocyte migration, and IGF transport) suppressed during active inflammation.

## References

[B1] Aibar S. González-Blas C. B. Moerman T. Huynh-Thu V. A. Imrichova H. Hulselmans G. (2017). SCENIC: single-cell regulatory network inference and clustering. Nat. Methods 14, 1083–1086. 10.1038/nmeth.4463 28991892 PMC5937676

[B2] Bhamidipati K. McIntyre A. B. R. Kazerounian S. Ce G. Wong S. W. Tran M. (2026). Spatial patterning of fibroblast TGFβ signaling underlies treatment resistance in rheumatoid arthritis. Nat. Immunol. 27, 556–571. 10.1038/s41590-025-02386-2 41540265 PMC12956583

[B3] Brand D. D. Kang A. H. Rosloniec E. F. (2004). The mouse model of collagen-induced arthritis. Methods Mol. Med. 102, 295–312. 10.1385/1-59259-805-6:295 15286392

[B4] Croft A. P. Campos J. Jansen K. Turner J. D. Marshall J. Attar M. (2019). Distinct fibroblast subsets drive inflammation and damage in arthritis. Nature 570, 246–251. 10.1038/s41586-019-1263-7 31142839 PMC6690841

[B5] Figueiredo M. L. (2024). Applications of single-cell RNA sequencing in rheumatoid arthritis. Front. Immunol. 15, 1491318. 10.3389/fimmu.2024.1491318 39600707 PMC11588722

[B6] Germain P.-L. Lun A. Garcia Meixide C. Macnair W. Robinson M. D. (2021). Doublet identification in single-cell sequencing data using scDblFinder. F1000Res 10, 979. 10.12688/f1000research.73600.2 35814628 PMC9204188

[B7] Guo Q. Wang Y. Xu D. Nossent J. Pavlos N. J. Xu J. (2018). Rheumatoid arthritis: pathological mechanisms and modern pharmacologic therapies. Bone Res. 6, 15. 10.1038/s41413-018-0016-9 29736302 PMC5920070

[B8] Isomäki P. Punnonen J. (1997). Pro- and anti-inflammatory cytokines in rheumatoid arthritis. Ann. Med. 29, 499–507. 10.3109/07853899709007474 9562516

[B9] Jagadesan S. Guda C. (2026). VST-DAVis: an R shiny application and web-browser for spatial transcriptomics data analysis and visualization. Bioinforma. Adv. 6, vbag007. 10.1093/bioadv/vbag007 41640624 PMC12866912

[B10] Jiang A. Snell R. G. Lehnert K. (2024). ICARUS v3, a massively scalable web server for single-cell RNA-seq analysis of millions of cells. Bioinformatics 40, 40. 10.1093/bioinformatics/btae167 38539041 PMC11007236

[B11] Jin S. Guerrero-Juarez C. F. Zhang L. Chang I. Ramos R. Kuan C.-H. (2021). Inference and analysis of cell-cell communication using CellChat. Nat. Commun. 12, 1088. 10.1038/s41467-021-21246-9 33597522 PMC7889871

[B12] Kehoe O. Cartwright A. Askari A. El Haj A. J. Middleton J. (2014). Intra-articular injection of mesenchymal stem cells leads to reduced inflammation and cartilage damage in murine antigen-induced arthritis. J. Transl. Med. 12, 157. 10.1186/1479-5876-12-157 24893776 PMC4053306

[B13] Kong J.-S. Jeong G. H. Yoo S.-A. (2023). The use of animal models in rheumatoid arthritis research. J. Yeungnam Med. Sci. 40, 23–29. 10.12701/jyms.2022.00773 36411592 PMC9946911

[B14] Korsunsky I. Millard N. Fan J. Slowikowski K. Zhang F. Wei K. (2019). Fast, sensitive and accurate integration of single-cell data with harmony. Nat. Methods 16, 1289–1296. 10.1038/s41592-019-0619-0 31740819 PMC6884693

[B30] Korsunsky I. Wei K. Pohin M. Kim E. Y. Barone F. Major T. (2022). Cross-tissue, single-cell stromal atlas identifies shared pathological fibroblast phenotypes in four chronic inflammatory diseases. Med 3, 481–518.e14. 10.1016/j.medj.2022.05.002 35649411 PMC9271637

[B31] Lilja S. Li X. Smelik M. Lee E. J. Loscalzo J. Marthanda P. B. (2023). Multi-organ single-cell analysis reveals an on/off switch system with potential for personalized treatment of immunological diseases. Cell Rep. Med. 4, 100956. 10.1016/j.xcrm.2023.100956 36858042 PMC10040389

[B15] Mashkoor M. Zhang S. Stensballe A. (2026). Bridging the precision gap in rheumatoid arthritis: spatial transcriptomics, spatial proteomics, and artificial intelligence in precision health. Biomedicines 14, 14. 10.3390/biomedicines14030668 41898314 PMC13024196

[B16] Muench D. E. Sun Z. Sharma A. Tang C. Crampton J. S. Lao C. (2022). A pathogenic Th17/CD38+ macrophage feedback loop drives inflammatory arthritis through TNF-α. J. Immunol. 208, 1315–1328. 10.4049/jimmunol.2101025 35197330

[B17] Nandakumar K. S. Holmdahl R. (2006). Antibody-induced arthritis: disease mechanisms and genes involved at the effector phase of arthritis. Arthritis Res. Ther. 8, 223. 10.1186/ar2089 17254316 PMC1794524

[B18] Rohini M. Haritha Menon A. Selvamurugan N. (2018). Role of activating transcription factor 3 and its interacting proteins under physiological and pathological conditions. Int. J. Biol. Macromol. 120, 310–317. 10.1016/j.ijbiomac.2018.08.107 30144543

[B19] Schett G. Gravallese E. (2012). Bone erosion in rheumatoid arthritis: mechanisms, diagnosis and treatment. Nat. Rev. Rheumatol. 8, 656–664. 10.1038/nrrheum.2012.153 23007741 PMC4096779

[B20] Szklarczyk D. Gable A. L. Lyon D. Junge A. Wyder S. Huerta-Cepas J. (2019). STRING v11: protein-protein association networks with increased coverage, supporting functional discovery in genome-wide experimental datasets. Nucleic Acids Res. 47, D607–D613. 10.1093/nar/gky1131 30476243 PMC6323986

[B21] Szklarczyk D. Gable A. L. Nastou K. C. Lyon D. Kirsch R. Pyysalo S. (2021). The STRING database in 2021: customizable protein-protein networks, and functional characterization of user-uploaded gene/measurement sets. Nucleic Acids Res. 49, D605–D612. 10.1093/nar/gkaa1074 33237311 PMC7779004

[B22] Tallack M. R. Whitington T. Yuen W. S. Wainwright E. N. Keys J. R. Gardiner B. B. (2010). A global role for KLF1 in erythropoiesis revealed by ChIP-seq in primary erythroid cells. Genome Res. 20, 1052–1063. 10.1101/gr.106575.110 20508144 PMC2909569

[B23] Torres A. Kang S. Mahony C. B. Cedeño M. Oliveira P. G. Fernandez-Bustamante M. (2023). Role of mitochondria-bound HK2 in rheumatoid arthritis fibroblast-like synoviocytes. Front. Immunol. 14, 1103231. 10.3389/fimmu.2023.1103231 37529037 PMC10389265

[B24] Trailmaker T. M. (2024). Parse Biosciences, Seattle, U.S.A. Available online at: http://app.trailmaker.parsebiosciences.com (Accessed 25 September, 2024).

[B25] Turner J. D. Filer A. (2015). The role of the synovial fibroblast in rheumatoid arthritis pathogenesis. Curr. Opin. Rheumatol. 27, 175–182. 10.1097/BOR.0000000000000148 25603041

[B26] Wei K. Korsunsky I. Marshall J. L. Gao A. Watts G. F. M. Major T. (2020). Notch signalling drives synovial fibroblast identity and arthritis pathology. Nature 582, 259–264. 10.1038/s41586-020-2222-z 32499639 PMC7841716

[B27] Zhang F. Jonsson A. H. Nathan A. Millard N. Curtis M. Xiao Q. (2023). Deconstruction of rheumatoid arthritis synovium defines inflammatory subtypes. Nature 623, 616–624. 10.1038/s41586-023-06708-y 37938773 PMC10651487

[B28] Zhou Y. Zhou B. Pache L. Chang M. Khodabakhshi A. H. Tanaseichuk O. (2019). Metascape provides a biologist-oriented resource for the analysis of systems-level datasets. Nat. Commun. 10, 1523. 10.1038/s41467-019-09234-6 30944313 PMC6447622

[B29] Zhu A. Ibrahim J. G. Love M. I. (2019). Heavy-tailed prior distributions for sequence count data: removing the noise and preserving large differences. Bioinformatics 35, 2084–2092. 10.1093/bioinformatics/bty895 30395178 PMC6581436

